# Revisiting Underwater Image Enhancement for Object Detection: A Unified Quality–Detection Evaluation Framework

**DOI:** 10.3390/jimaging12010018

**Published:** 2025-12-30

**Authors:** Ali Awad, Ashraf Saleem, Sidike Paheding, Evan Lucas, Serein Al-Ratrout, Timothy C. Havens

**Affiliations:** 1 Department of Applied Computing, College of Computing, Michigan Technological University, Houghton, MI 49931, USA; 2 Department of Computer Science and Engineering, Fairfield University, Fairfield, CT 06824, USA; 3 Department of Computer Science, Michigan Technological University, Houghton, MI 49931, USA

**Keywords:** underwater imaging, image enhancement, object detection, image quality assessment, per-image evaluation, mAP analysis, enhancement–detection interaction

## Abstract

Underwater images often suffer from severe color distortion, low contrast, and reduced visibility, motivating the widespread use of image enhancement as a preprocessing step for downstream computer vision tasks. However, recent studies have questioned whether enhancement actually improves object detection performance. In this work, we conduct a comprehensive and rigorous evaluation of nine state-of-the-art enhancement methods and their interactions with modern object detectors. We propose a unified evaluation framework that integrates (1) a distribution-level quality assessment using a composite quality index (Q-index), (2) a fine-grained per-image detection protocol based on COCO-style mAP, and (3) a mixed-set upper-bound analysis that quantifies the theoretical performance achievable through ideal selective enhancement. Our findings reveal that traditional image quality metrics do not reliably predict detection performance, and that dataset-level conclusions often overlook substantial image-level variability. Through per-image evaluation, we identify numerous cases in which enhancement significantly improves detection accuracy—primarily for low-quality inputs—while also demonstrating conditions under which enhancement degrades performance. The mixed-set analysis shows that selective enhancement can yield substantial gains over both original and fully enhanced datasets, establishing a new direction for designing enhancement models optimized for downstream vision tasks. This study provides the most comprehensive evidence to date that underwater image enhancement can be beneficial for object detection when evaluated at the appropriate granularity and guided by informed selection strategies. The data generated and code developed are publicly available.

## 1. Introduction

Underwater computer vision has become increasingly important in ecological monitoring, habitat mapping, and autonomous inspection tasks enabled by advances in Remotely Operated Vehicles (ROVs) and Autonomous Underwater Vehicles (AUVs). The availability of large underwater datasets [1,2,3]—many of which are fully or partially collected using ROVs due to their low cost and ease of deployment—has accelerated research in this area. However, underwater images are often degraded by absorption and scattering processes [4], resulting in reduced contrast, color shifts, and low visibility. These degradations negatively impact both human visual interpretation and the performance of downstream high-level tasks.

To address these challenges, numerous underwater image enhancement (UIE) approaches have been introduced, spanning non-physical methods [5], physics-based models [6], and learning-based techniques [7,8]. While it is often assumed that enhancement improves subsequent tasks, prior studies report conflicting conclusions. Some works observed that enhancement can support classification or detection when used as a preprocessing step [9,10], while others found that enhancement does not recover performance lost due to degradation [11] or may even reduce detection accuracy [12,13]. Saleem et al. [14] further showed that mixing enhanced and non-enhanced images may improve robustness but not absolute performance.

Despite these efforts, current evaluations of enhancement–detection interactions exhibit two key limitations. First, existing studies predominantly rely on *dataset-level* metrics, such as mean Average Precision (mAP), to assess detection performance. These aggregated metrics hide image-specific behavior, making it impossible to determine which images benefit from enhancement and which are adversely affected. Second, conventional underwater image-quality metrics (e.g., UIQM, UCIQE, CCF, Entropy) assess quality on a per-image basis, but do not reveal how enhancement redistributes quality across an entire dataset. As a result, enhancement is treated as a uniform transformation, even though low-quality images may improve, while high-quality images may become over-enhanced or distorted. These inconsistencies are further reinforced by recent findings showing that underwater color reconstruction is inherently ambiguous and physically non-unique [15]. Such results suggest that appearance-based metrics alone cannot fully characterize enhancement quality, especially when the goal is to support downstream vision tasks such as object detection.

These limitations prevent a fine-grained understanding of how enhancement affects detection performance, particularly at the level where detection decisions occur: the individual image. They also hinder the development of selective or adaptive enhancement strategies tailored to the characteristics of specific images.

To address these gaps, we propose a comprehensive evaluation framework for studying the relationship between underwater image enhancement and object detection at both dataset and image levels. The key components of this work are as follows:

- A unified quality-index representation (Q-index) that combines four established no-reference underwater image-quality metrics through global normalization and simple equal-weight aggregation. The Q-index is not introduced as a new image-quality metric; instead, it serves as an analytical tool for modeling how enhancement shifts the quality distribution of entire datasets and for interpreting quality–detection interactions without bias toward any single metric.
- A per-image mAP evaluation protocol that adapts COCO-style mAP calculations to individual images. This protocol enables precise, image-level attribution of detection performance changes after enhancement—a capability not supported by standard dataset-level evaluations.
- A mixed-set upper-bound analysis in which, for each image, the variant (original or enhanced) with the highest per-image mAP is selected. This analysis quantifies the maximum achievable improvement through selective enhancement and reveals performance gains that are obscured by dataset-level averages.
- An extensive experimental study using nine UIE models, three object detectors, and two public underwater datasets. Unlike prior work, our evaluation emphasizes distribution-level and image-level reasoning, highlighting when and why enhancement helps or harms detection.

Our results show that enhancement reliably improves low-quality images while often over-enhancing high-quality ones, altering dataset-quality distributions. We further demonstrate that improvements in conventional quality metrics do not necessarily correlate with detection performance. Most importantly, our per-image analysis reveals that a substantial subset of images achieves better detection performance after enhancement—a phenomenon masked by dataset-level metrics. These findings underscore the need for selective enhancement strategies and establish a principled foundation for understanding enhancement–detection interactions in underwater remote sensing.

The remainder of this work is organized as follows. Section 2 surveys recent enhancement–detection studies, state-of-the-art underwater image enhancement models, and underwater object detection models. Section 3 details the experimental setup and evaluation procedures. Image enhancement results are analyzed in Section 4, and object detection results are examined in Section 5. The core analysis of this study, including the per-image evaluation, mixed-set construction, and upper-bound interpretation is provided in Section 6. Finally, Section 7 concludes the paper.

## 2. Literature Survey

In this section, we review combined enhancement–detection studies, underwater image enhancement (UIE) models, and Underwater Object Detection (UOD) models.

### 2.1. Combined Enhancement–Detection Studies

A number of studies addressed the effect of image enhancement on object detection. For instance, the authors of [16] studied the effect of mixed domains and different environments on detection and discussed the enhancement role in the process. The authors concluded that image restoration does not increase within-domain detection performance, but rather produces a more generalizable performance. One of the limitations of the study presented in [16] is that it is limited to a single restoration technique. In a related effort, Pei et al. [11] examined the effect of synthetic image degradation and restoration on classification performance. This study concluded that although image degradation severely affects the classification performance, image enhancement (i.e., degradation removal) does not lead to a restored or improved performance. This study can be extended to other higher-level tasks such as object detection. In another study, Ref. [17] introduced a new video dataset called Underwater Visual Object Tracking (UVOT) and an enhancement model designed to improve object tracking performance. The study claimed significant detection performance improvement. However, no statistical significance analysis was provided in the study to validate the authors’ claimed improvement. Another group of researchers [13] further questioned the effect of enhancement on detection. The authors used the Toolbox for Identifying Object Detection Errors (TIDE) [18] to conduct their analysis, which revealed that enhancement increases the false positive (FP) rate because it changes the objects’ edges that are essential for detection. In a recent development in image enhancement and object detection, Fu et al. [12] proposed a new method for combining image enhancement and object detection using a shared loss function to train both the enhancer and detector simultaneously. The findings of their research indicate that the traditional enhancement approach of preprocessing the images with enhancement leads to a lower detection performance, while a joint-learning approach increases it. The authors do not provide many details about the implementation of their proposed joint-learning framework. Moreover, the authors in [19] introduced a novel enhancement–detection joint-learning framework based on reinforcement learning (RL). An RL agent is trained on the URPC2018 [20] dataset with an action space consisting of a group of enhancement effects, a state space represented by image features, and detection performance as a reward. The authors claimed an improvement over the traditional preprocessing approach. This work could be expanded by extending the action space to include more enhancement effects and algorithms.

### 2.2. Underwater Image Enhancement (UIE)

Underwater image enhancement is categorized into three main groups [21,22,23]: non-physical, physical, and learning-based methods. In general, non-physical models work directly on images using image processing techniques without using any prior knowledge, so they are fast and simple but often lead to sub-optimal enhancement details [24]. For instance, Zhang et al. [5] introduced a method for enhancing underwater images using a combination of various techniques. Their proposed method included attenuated color channel correction (ACCC), a fusion-based contrast improvement method, and a multiscale unsharp masking (MSUM) strategy. Although this method generalizes well to low-light images and hazy images, it may reduce the color quality of already low-quality images. This method is also computationally expensive. The retinex-based enhancement model in [25] used multi-order gradient priors of reflectance and illumination. This approach is based on a simple and effective color correction approach to remove color casts and recover naturalness. Then a maximum posterior formulation is used by imposing multi-order gradient priors on both reflectance and illumination. Another model called Texture Enhancement Model based on Blurriness and Color Fusion (TEBCF) is presented in [26]. This model used multi-scale fusion to merge two inputs; one to improve contrast based on dark channel prior in the RGB space and another to improve color based on the CIELAB color space [27]. Although the method introduced in [26] showed a significant quantitative performance advantage over other approaches, it did not perform as well qualitatively.

In contrast to the non-physical methods described, physical methods incorporate some kind of prior knowledge based on the physics of the underwater image formation process. However, the assumptions made for unknown parameters may not be entirely suitable or precise in complex underwater environments, which restricts their use cases [28]. For example, Zhang et al. [6] developed an approach that begins with a color cast correction method for each color channel. Subsequently, various enhancement methods are applied to enhance the base and detail layers of the V channel in the HSV space, which is decomposed using the spatial prior and texture prior. This method showed good generalization capability for fog and low-light images. In another enhancement approach [29], a new model called Illumination Channel Sparsity Prior (ICSP) was developed based on the observation that there are some low-intensity pixels in the illumination (I) channel of an underwater image with uniform lighting in the HSI color space. Using this observation, the authors developed a variational model based on the retinex theory [30]. However, some images produced by this model are over-brightened.

Unlike both physical and non-physical enhancement methods, learning-based enhancement methods rely on neural networks and can generate high-quality enhancement results but require parameter tuning and a sufficient amount of data [31], which can be challenging for underwater environments. Researchers in [32] introduced a Mean Teacher-based Semi-supervised Underwater Image Restoration (Semi-UIR) model which incorporates unlabeled data into network training. Similarly, Fu et al. [33] introduced an UnSupervised Underwater Image Restoration (USUIR) method by leveraging the homology property between a raw underwater image and a ’re-degraded’ image. This proposed approach decomposes the underwater image into three latent components. The design of this model allows it to run relatively fast and at the same time achieve good qualitative results. In [34], a Two-phase Underwater Domain Adaptation network (TUDA) was introduced, which simultaneously minimizes the inter-domain and intra-domain gaps. First, a triple-alignment network was designed to jointly perform image-level, feature-level, and output-level alignment using adversarial learning to reduce the inter-domain gap. Then, an easy/hard adaptation technique was developed to reduce intra-domain gaps. The colors produced by this method are vibrant and the images are visually pleasing. Another group of researchers [7] applied Neural Architecture Search (NAS) to search for the optimal U-Net architecture specifically tailored for underwater image enhancement. This approach demonstrated good visual performance across different underwater scenarios. A different approach was explored in [35], where underwater image enhancement was modeled as a Markov decision process (MDP). This approach was based on reinforcement learning frameworks that selected a set of image enhancement actions and organized them into an optimal sequence. This is one of the preliminary studies on the use of reinforcement learning in underwater image enhancement.

### 2.3. Underwater Object Detection (UOD)

Object detection algorithms can be roughly categorized into one-stage and two-stage detectors. One-stage detectors generate object coordinates and labels in a single step, making them faster but generally less accurate. In contrast, two-stage detectors use a separate region proposal network to first identify potential object locations before classifying the objects, resulting in higher performance at the cost of speed. A group of researchers developed two-stage detectors for underwater scenarios. For example, Mandal et al. [36] integrated the Faster R-CNN [37] with three distinct backbone networks to detect fish species. In another work, Lin et al. [38] introduced a data augmentation technique that used candidate region fusion to create training samples that mimic overlap, occlusion, and blurring effects. Xu et al. [39] developed a scale-aware feature pyramid detector based on a unique backbone sub-network which captures the fine-grained features of smaller targets. Qi et al. [40] introduced a detection model based on a deformable convolutional pyramid structure to identify small underwater objects. Song et al. [41] proposed a new model comprised of a region proposal network that provided the prior probability of objects, then the classification score and the prior uncertainty were combined to generate the score of the final prediction. Finally, the classification loss is increased for miscalculated proposals, whereas it is reduced for accurately predicted proposals.

Other works have focused on one-stage underwater detectors. For instance, Sung et al. [42] focused on detecting fish in real time using a YOLO-v1 [43]-based detection framework. Similarly, Hu et al. [44] developed a detection network for sea urchins based on the detection algorithm presented in [45]. A novel multi-directional edge detection technique was introduced to better capture the distinctive spiny edge characteristics of sea urchins, thereby improving feature representation. Chen et al. [46] introduced a neural network architecture called SWIPENet based on the framework presented in [47]. This model was designed for the detection of small targets in underwater environments. Furthermore, the YOLO architecture has been used in several works. For instance, Zhang et al. [48] introduced a lightweight approach for recognizing underwater objects based on YOLO-v4 [49]. Liu et al. [50] proposed TC-YOLO, a novel underwater object detection technique that includes attention mechanisms by integrating a coordinate attention module and a transformer encoder into YOLO-v5 [51]. YOLO-NAS [52] introduced a novel algorithmic optimization engine to the YOLO family known as Automated Neural Architecture Construction (AutoNAC), which guarantees optimal hardware utilization while maintaining baseline performance.

## 3. Evaluation Framework and Experimental Setup

**Figure 1:**
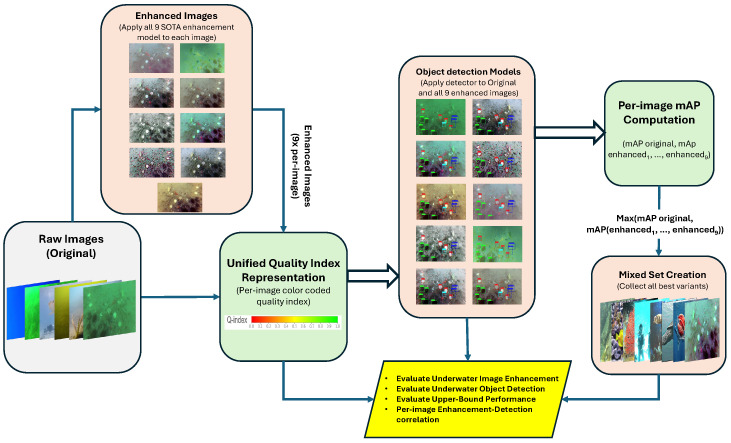
Overview of the proposed evaluation framework. Raw images are enhanced using nine UIE models, evaluated through a unified Q-index, and processed by object detectors to compute per-image mAP. The best-performing version of each image forms a mixed set used to estimate the upper-bound detection performance. Green blocks denote methods newly proposed in this work, peach blocks indicate existing methods re-implemented in this study, and yellow blocks represent result evaluation, interpretation, and analytical insights.

This section presents the proposed evaluation framework used to analyze the interaction between underwater image enhancement and object detection. As illustrated in Figure 1, the framework begins with the raw underwater images, which are processed by nine state-of-the-art enhancement models to generate multiple enhanced variants. Both the original and enhanced images are then evaluated using four established no-reference image quality metrics, which are combined through global normalization into a unified quality-index representation (Q-index) to support distribution-level quality analysis. In parallel, the original and enhanced images are passed through the object detection models, and a per-image mAP score is computed for each version of every image. By selecting the version that yields the highest per-image mAP, we construct a mixed set containing the best-performing variant for each image. Evaluating this mixed set provides an upper-bound estimate of the potential performance gains achievable through selective enhancement. Together, these components enable a comprehensive assessment of underwater image enhancement, underwater object detection, upper-bound performance, and the relationship between per-image quality and detection outcomes.

### 3.1. Underwater Image Enhancement Models

To support our study, we selected representative image enhancement methods that cover the main enhancement paradigms. The non-physical methods include ACDC [5], TEBCF [26], and BayesRet [25], covering histogram-based, fusion-based, and Retinex-based enhancement, respectively. The physical-model-based methods include PCDE [6] and ICSP [29], representing dark channel prior approaches and optical imaging property models. The deep learning methods include AutoEnh [7], Semi-UIR [32], USUIR [33], and TUDA [34], covering transformer-based, contrastive-learning-based, and GAN-based frameworks. All enhancement models were used exactly as provided by their original authors, including source code and trained weights, with no modifications or retraining.

### 3.2. Object Detection Models

For object detection, we selected three widely used architectures: YOLO-NAS [52], RetinaNet [53], and Faster R-CNN [53], covering both one-stage and two-stage paradigms. The top-performing detector on the original dataset was used for subsequent evaluations. For each enhancement method and each dataset, a separate detector was trained, resulting in 20 independently trained model instances.

Following the RUOD authors [12], all images were resized to 800×600 without padding. YOLO-NAS was implemented using the SuperGradients library [52], whereas RetinaNet and Faster R-CNN were implemented using Detectron2 [53]. All experiments were conducted on a Linux server equipped with two NVIDIA Tesla V100 GPUs. For YOLO-NAS, we used the Large (L) variant with COCO pre-trained weights, a batch size of 16, and the AdamW optimizer with a weight decay of $1\times 10^{-5}$. Training ran for 300 epochs with a learning rate starting at $2\times 10^{-4}$ and a linear warmup for the first 1000 steps from $1\times 10^{-6}$, followed by cosine decay. Default Mosaic augmentation was used. During testing, we used a confidence threshold of 0.001 and an NMS threshold of 0.7. RetinaNet and Faster R-CNN used Detectron2 model-zoo defaults. Learning rates were 0.02 for Faster R-CNN and 0.01 for RetinaNet, with a batch size of 16. A multi-step scheduler with a milestone at 30,000 iterations and decay factor 0.1 was used. Detectron2’s default data augmentation (random horizontal flip, p=0.5) was applied. During testing, thresholds were set to 0.05 for confidence and 0.5 for NMS.

To ensure reproducibility and consistency across experiments, each object detector was trained independently for each dataset and enhancement condition using a consistent training protocol. YOLO-NAS, RetinaNet, and Faster R-CNN were initialized with COCO-pretrained weights provided by their official implementations and fine-tuned on the corresponding underwater training sets. Detector architectures, optimizers, learning rates, batch sizes, training schedules, and inference thresholds were kept fixed across enhancement conditions, with only the input images differing.

### 3.3. Datasets

We evaluate on two publicly available underwater datasets to ensure broad generalizability: CUPDD [54] and RUOD [12]. RUOD is one of the largest available underwater datasets, combining images from multiple sources and containing diverse color casts, environments, and aquatic species. It contains 14,000 high-resolution images, of which 9800 are used for training, with approximately 75,000 bounding box annotations across ten classes. CUPDD is smaller but significantly more challenging, containing 414 images (313 for training) with three general categories of aquatic plants from the Great Lakes region. The contrasting characteristics of the two datasets enable robust analysis across large-scale and heavily degraded scenarios.

### 3.4. Unified Quality-Index Representation (Q-Index)

Because ground-truth reference images without underwater degradation are not available, we evaluate image quality using four established no-reference metrics: UIQM [55], UCIQE [56], CCF [57], and Entropy [58]. Each metric measures a different aspect of underwater visibility, including colorfulness, contrast, sharpness, and information content. To support distribution-level analysis, we combine these four metrics into a unified quality-index representation (Q-index). The Q-index is not a new image-quality metric; rather, it provides a normalized, aggregated indicator used solely to visualize and interpret how enhancement redistributes quality across datasets. The Q-index is constructed in three steps:

1. Outlier removal: Values more than three Median Absolute Deviations (MADs) from the median are removed using the MATLAB R2025b method in [59].
2. Global rescaling: Each quality metric is min–max normalized across all original and enhanced images so that its values fall within [0,1].
3. Equal-weight aggregation: The four normalized metrics are averaged to produce a single bounded representation. Equal weighting avoids bias toward any individual metric and is appropriate given the absence of ground-truth reference images that would justify optimized or data-driven weighting. We consider ±25% weight perturbations as a reasonable sensitivity range to assess whether aggregated quality trends could be influenced by a single component metric; however, in this study the Q-index is used strictly as an interpretive tool for distribution-level analysis rather than as a statistically optimized quality measure.

### 3.5. Per-Image Detection Evaluation and Mixed-Set Upper Bound

For each image in the dataset, we run the detector on both the original image and all nine enhanced variants. Instead of combining predictions from the entire dataset, we adapt the COCO protocol to compute precision–recall curves for *each individual image*. Specifically, detections for a single image are matched to its ground-truth annotations using IoU thresholds from 0.50 to 0.95 in increments of 0.05. The resulting average precision values are then averaged across IoU thresholds, producing a per-image mAP_50:95_ score for every image–enhancer pair. This per-image evaluation is essential because enhancement may improve detection for some images while degrading it for others. These heterogeneous effects are completely obscured by dataset-level metrics, which average performance across all images.

Using the per-image mAP scores, we construct a *mixed-set upper bound* to quantify the maximum performance achievable by a hypothetical selective enhancement strategy. For each image, we compare the per-image mAP of the original image with that of all enhanced versions and retain the version (original or enhanced) that achieves the highest score. Formally, let each image *n* have *v* enhanced versions in addition to the original. Denote the per-image mAP of the *v*-th version by ${\mathrm{mAP}}_{n,v}$, where v=0 corresponds to the original image. For each image, we select the most favorable version:

(1) $$v_{n}^{*}=\arg\max_{v\in \{0,\ldots ,V\}}{\mathrm{mAP}}_{n,v}$$

(2) $$S_{\mathrm{mix}}=\left\{\left(n,v_{n}^{*}\right):n=1,\ldots ,N\right\}$$

(3) $${\bar{\mathrm{mAP}}}_{\mathrm{mix}}=\frac{1}{N}\sum_{n=1}^{N}{\mathrm{mAP}}_{n,v_{n}^{*}}$$

Collecting these best-performing versions across all images yields a mixed set that represents the theoretical upper bound on detection performance. Evaluating the detector on this mixed set provides an upper-bound estimate of what could be achieved if an oracle were able to choose, for each image, whether to apply enhancement and which enhancement model to use. While this is not a deployable system, it quantifies the latent potential of enhancement techniques and demonstrates that significant improvements may be achievable through future adaptive or learned selection mechanisms. Together, the image-level mAP protocol and the mixed-set upper-bound analysis offer a detailed and nuanced view of how enhancement interacts with detection at the image level. They reveal not only which images benefit from enhancement but also the magnitude of improvements that dataset-level metrics fail to capture.

## 4. Enhancement Evaluation

Following the proposed evaluation framework, this section analyzes how underwater image enhancement affects image quality using the four no-reference image-quality metrics and the unified Q-index representation. Unlike prior work that focuses only on dataset-level averages, this analysis establishes the quality patterns that later relate to per-image detection outcomes and the mixed-set upper-bound analysis (Section 6.1). A quantitative and qualitative evaluation of the selected image enhancement models is presented for both the RUOD and CUPDD datasets. In addition, we generate the Q-index based on the selected metrics to show the quality distribution of the images after enhancement and to provide a joint quantitative and qualitative analysis.

### 4.1. Quantitative Enhancement Evaluation

The images from the selected datasets are enhanced using the selected enhancement models and evaluated using the selected image quality metrics (UIQM, UCIQE, CCF, and Entropy). These four metrics collectively form the basis of the unified Q-index used in the distribution-level analysis presented in Section 4.2. All metric values are globally rescaled, as described in Section 3, to ensure fair comparison across all enhancement models and datasets. On the one hand, the results for the CUPDD dataset are shown in Table 1. These results show the high performance of the TEBCF model [26] based on all metrics, especially in terms of the UIQM valued at 4.07 and CCF valued at 25.03. This should indicate its capability of producing vibrant colors, marked contrast, soft edges, and removing fog effects. The Semi-UIR model also performs notably with a UIQM of 3.21 and high UCIQE and Entropy values (0.55 and 7.56, respectively), suggesting that it offers a balanced image quality enhancement with rich details. Meanwhile, the USUIR model stands out in UCIQE (0.60) and Entropy (7.67), reflecting its proficiency in improving colorfulness and retaining image detail. The ACDC is another notable model that signifies a marked enhancement capability with a UIQM of 3.07 and balanced performance on other metrics. Similarly, BayesRet and TUDA demonstrate a balanced approach with a commendable Entropy at 7.72 and 7.77, respectively, indicating substantial detail retention alongside reasonable color and contrast enhancements. Conversely, models like ICSP and PCDE, with lower UIQM and CCF values, reflect limited capabilities in quality enhancement and color correction. These comparative insights highlight the strengths and potential applications of each model in underwater image processing, emphasizing the importance of selecting a model based on the desired balance of quality, color correction, and detail retention. It is worth noting that all models are performing better than the original except for the ICSP, indicating that it is possibly distorting colors and introducing noise. The PCDE and the BayesRet are also less effective than the original, indicating their limited capabilities of enhancing underwater images.

On the other hand, the results for the RUOD dataset are shown in Table 2. These results show relatively higher CCF and Entropy scores compared to the results on the CUPDD dataset. This could be attributed to the fact that RUOD contains clearer images with richer details. In a similar performance jump, the TEBCF [26] is a top-performer in terms of the UCIQE and the CCF at 0.62 and 31.5, respectively. The TEBCF is followed by the BayesRet at top UIQM and entropy values of 3.85 and 7.74, respectively. This might indicate the capability of the TEBCF in removing the fog effect and the capability of the BayesRet in adding more details. The ACDC also shows robust performance with a high UIQM (3.76) and balanced metrics, rendering it a versatile candidate for underwater image enhancement. TUDA further demonstrates solid capability with a UIQM of 3.40, a UCIQE of 0.58, a CCF of 20.88, and a high Entropy of 7.65, highlighting it as a strong performer overall, particularly excelling in maintaining detail. Models like AutoEnh and Semi-UIR are notable for their good colorfulness and contrast enhancement at a UCIQE of 0.59 and 0.60, respectively, with reasonable overall quality and detail retention. In contrast, PCDE and ICSP exhibit lower UIQM (2.42 and 1.14) and CCF (14.76 and 25.59), reflecting their relatively less impactful enhancements. Aligning with the results from Table 1, all models outperform the original except for the ICSP and the PCDE on some metrics, reflecting the limited capabilities of both models.

### 4.2. Quality Distribution

**Figure 2:**
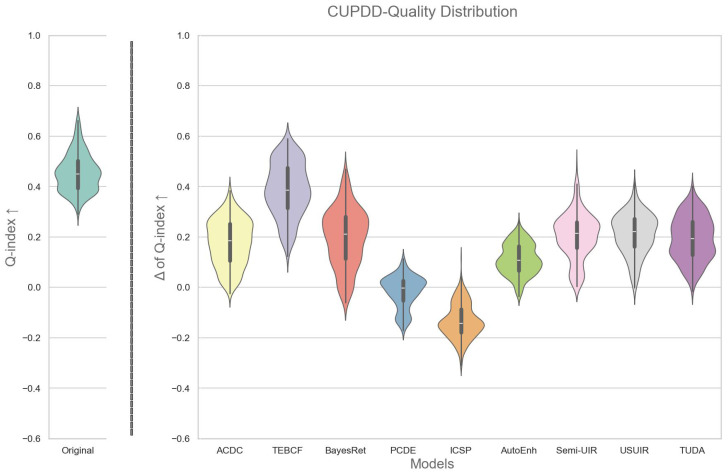
On the left, the quality distribution of the original images of the CUPDD dataset [54] based on the Q-index. On the right, the distributions of the change in quality after enhancement by different models.

**Figure 3:**
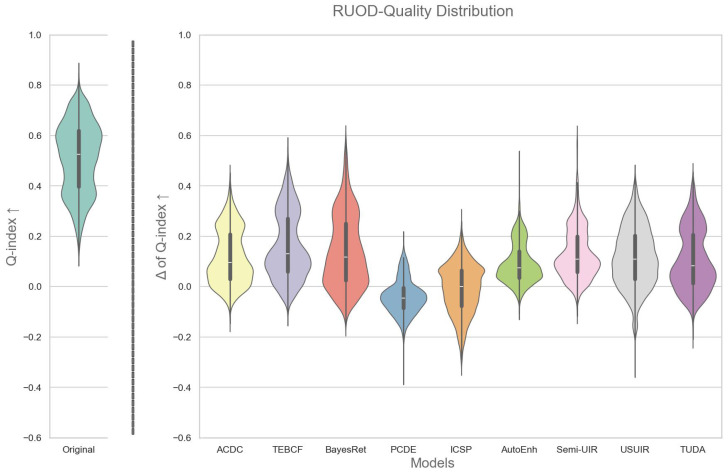
On the left, the quality distribution of the original images of the RUOD dataset [12] based on the Q-index. On the right, the distributions of the change in quality after enhancement by different models.

We used the proposed Q-index in Section 3 to facilitate an analysis of the quality distribution of images before and after enhancement by each enhancement model. In particular, a violin plot is used to show the quality distribution of original images and the change in the quality distribution after enhancement, as shown in Figure 2 and Figure 3. The change in the Q-index (ΔQ-index) for an image is simply calculated as

(4) $$\begin{array}{c} \begin{array}{c} \Delta \;\;\text{Q-index}={\text{Q-index}}_{\left(Enh\right)}-{\text{Q-index}}_{\left(Org\right)}, \end{array} \end{array}$$

where (Enh) refers to the enhanced image and (Org) refers to the original. This helps us understand how enhancement is changing the quality distribution of the images, i.e., whether enhancement uniformly improves the quality of images across the quality spectrum. On the one hand, by examining Figure 2, it is observed that the entire original image dataset ranges from 0.25 to 0.75, with an almost bimodal distribution and a median of approximately 0.45. One peak is centered around the median, and the other is at just below 0.4, indicating that most images lie around those two peaks. Moving to the distribution of images after enhancement, we notice that all models, except for the PCDE and ICSP, produced a positive distribution. The PCDE, with almost half the distribution in the negative, and ICSP, with the entire distribution in the negative, generally produce images that have lower quality than the originals. TEBCF is a top performer at a median of approximately 0.38, followed by ACDC, BayesRet, Semi-UIR, USUIR, and TUDA at a median of approximately 0.2. In contrast, AutoEnh showed lower performance, exhibiting a bimodal distribution with most of the images centered around 0.1. It is also noted that for ACDC, TEBCF, BayesRet, Semi-UIR, and USUIR, the distribution has peaks at the top and tapers off towards the bottom, indicating that these models are shifting low-quality images into high-quality images. A small number of images performed worse after enhancement with the ACDC, TEBCF, AutoEnh, Semi-UIR, USUIR, and TUDA models. This distribution-level analysis is essential for understanding when enhancement is beneficial or detrimental, and it directly supports the mixed-set construction.

On the other hand, the distribution of the original RUOD images shown in Figure 3 is a clearer bimodal distribution with a median of nearly 0.52 and two distinct peaks at 0.6 and 0.35. Furthermore, most enhancement models have a median at 0.1 except for the PCDE and ICSP, which exhibit similar negative trends as in Figure 2. A notable observation about the distribution of the enhanced images in Figure 3 is that most models have an inverted distribution compared to the original with a larger peak at the bottom. This means that enhancement is deteriorating the quality of high-quality images by over-enhancing them in the RUOD dataset. On the contrary, CUPDD had the larger peak on top. This could be attributed to the fact that CUPDD images are mostly severely degraded images and have significantly lower quality than RUOD. This supports that enhancement is more beneficial to low-quality images.

### 4.3. Joint Quantitative–Qualitative Enhancement Evaluation

**Figure 4:**
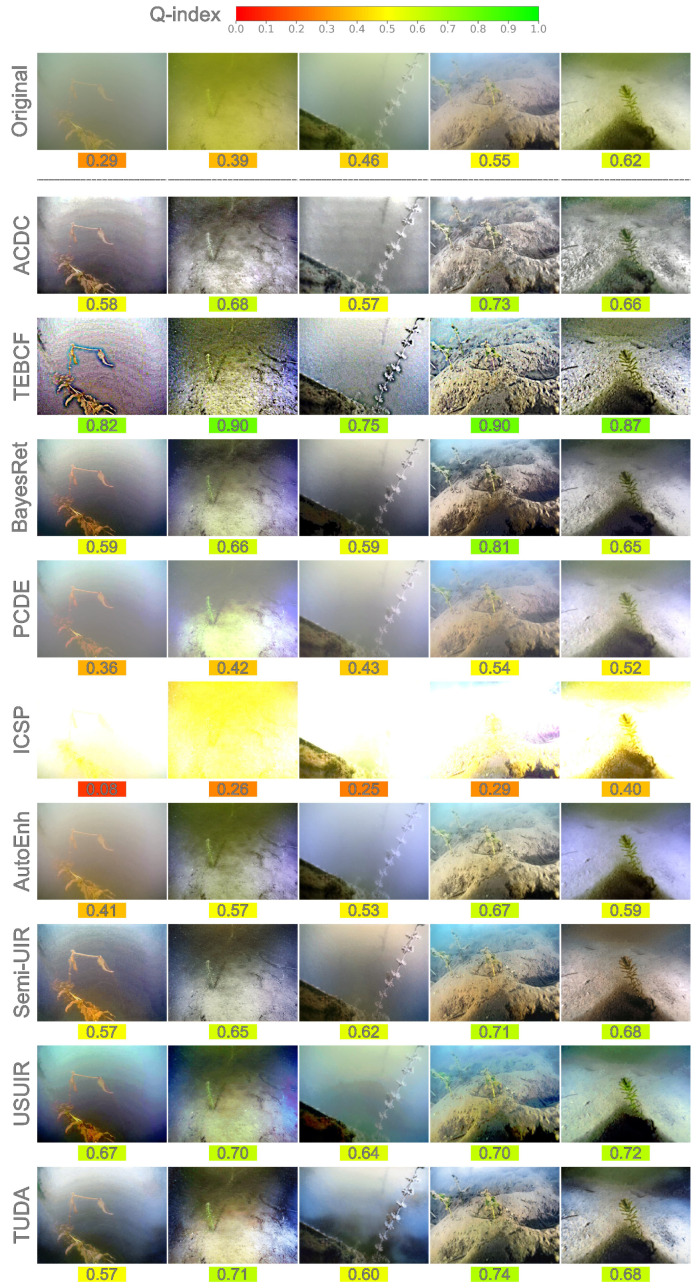
Randomly selected original images from each available quality bin of the CUPDD dataset. The corresponding Q-index values are color-mapped and placed under each image.

**Figure 5:**
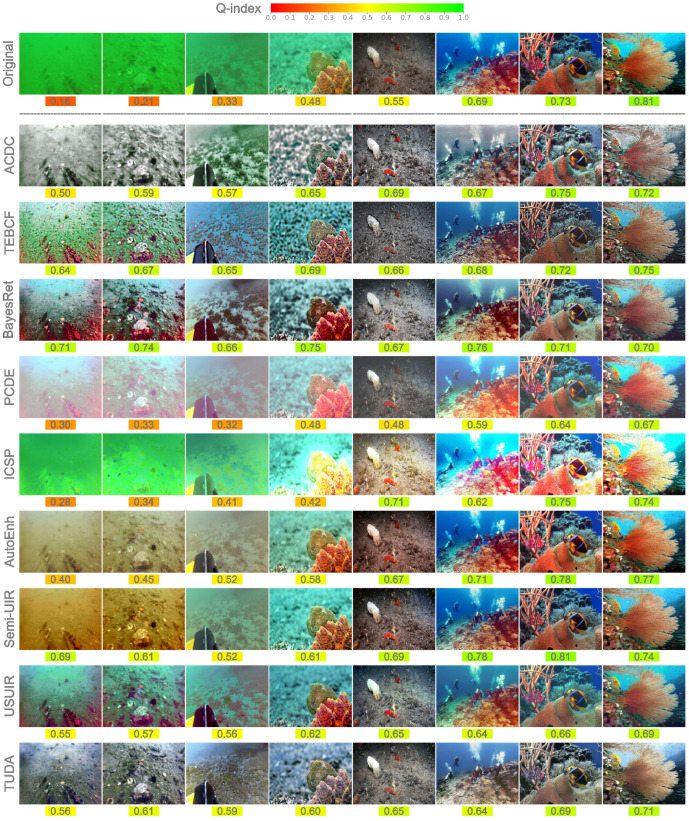
Randomly selected original images from each available quality bin of the RUOD dataset. The corresponding Q-index values are color-mapped and placed under each image.

As a further and final evaluation of image enhancement, we categorize images into 10 quality bins (levels) based on their Q-index values and randomly select an image representing each quality bin for both datasets, namely the CUPDD and the RUOD as shown in Figure 4 and Figure 5. Some quality bins at both ends are omitted since no images are found in those extreme bins. The Q-index value is shown under each image and color-mapped from red (low-quality) to green (high-quality) for easier perception of the quantitative quality. Figure 4 shows a random image from each quality bin available in the CUPDD dataset with its enhanced versions and corresponding Q-index values. A gradual improvement in quality can be seen as the Q-index value of the original images increases from left to right. However, we notice that the image belonging to the 0.5–0.6 quality bin looks more visually pleasing than its successor with softer details, less noise, and more vibrant colors. The successor original image at a higher Q-index of 0.62 displays a stronger color cast and more noise, yet achieves a higher Q-index, which questions the reliability of the current underwater image quality metrics. This observation is further demonstrated by the enhanced images, especially with the TEBCF images, where they all look very sharp and noisy but achieve Q-index values exceeding 0.75. In contrast, the Q-index aligns well with our qualitative evaluation of the PCDE and the ICSP models at values ranging from 0.52 to values as low as 0.08. PCDE produces very foggy images, whereas ICSP produces extremely overexposed images, further deteriorating the quality of the original images. This suggests that although current underwater metrics are not entirely robust, they can still provide a general indication of image quality. ACDC and BayesRet produce less saturated color tones but maintain the details of the images. AutoEnh, Semi-UIR, USUIR, and TUDA seem to be producing the most visually pleasing images with vibrant colors, less noise, and fog effects compared to other models. However, this is not always reflected by their Q-index values. Notably, the average increase in the Q-index value of low-quality images is much higher than the average increase in the Q-index values of high-quality images. For instance, the Q-index of the image from the lowest quality bin nearly doubled with some models, whereas the Q-index of the image from the highest quality bin showed only a marginal improvement with most models. In fact, most high-quality original images in the RUOD Figure 5 attained lower Q-index values after enhancement. This supports the conclusion from our quality distribution analysis in Section 4.2 that enhancement of high-quality images often results in over-enhancement.

In accordance with CUPDD, the random images from the RUOD dataset shown in Figure 5 exhibit a similar gradual improvement from left to right. Images with a low Q-index tend to have a monochromatic cast with very few details and low visibility. A less severe color cast with more details and colors can be noted as the Q-index increases. Images with a high Q-index tend to have a richer red channel, more vibrant color, and clearer fine details. Although it is hard to distinguish a slight variation in the Q-index, the general trend of quality improvement with the increase in the Q-index is evident. For instance, the quality of the first two images at 0.16 and 0.21 is hardly distinguishable, but the difference between the first and last images is highly notable. The performance of the enhancers on RUOD is very similar to CUPDD with TEBCF quantitatively leading but qualitatively lagging behind. This discrepancy in the quantitative and qualitative further highlights the limitation of current underwater metrics. We identify TUDA and Semi-UIR as the best enhancers for producing visually pleasing images. We conclude that enhancement techniques are most effective on low-quality images and less effective on high-quality images. Moreover, we observe that the selected deep learning-based enhancement models produce more visually pleasing images than traditional models. Lastly, current underwater image quality metrics can be used as a general indication of image quality, despite their limitations. These qualitative observations complement the per-image mAP evaluation in Section 6.1, enabling a direct comparison between visual quality changes and their impact on detection accuracy. Overall, the enhancement-induced quality variations identified in this section provide the foundation for the per-image detection analysis in Section 6.1 and the upper-bound performance estimation in Section 6.2.

## 5. Detection Evaluation

Building on the enhancement analysis in Section 4, this section presents the quantitative and qualitative evaluation of the underwater object detection models when trained and tested on the original and enhanced datasets. The focus here is exclusively on dataset-level detection performance, establishing how enhancement affects overall mAP trends. Image-level variations in detection accuracy, including per-image mAP computation and mixed-set construction, are presented separately in Section 6.

In this section, we first compare the detection performance of the selected three detection algorithms on the original images as shown in Table 3, where YOLO-NAS outperformed all other algorithms by a large margin, followed by RetinaNet, and finally Faster R-CNN. Therefore, YOLO-NAS is selected as the best-performing detector, and a total of 20 models are created from YOLO-NAS, including nine models for the enhanced versions of CUPDD, nine models for the enhanced versions of the RUOD, and two models for the original images of CUPDD and RUOD. The YOLO detection family has been extensively used in underwater scenarios, demonstrating its efficiency [43,48,50] with YOLO-NAS being one of the recent top performers [60,61,62].

While dataset-level metrics provide important insight into global performance trends, they do not fully capture how enhancement affects individual images. Therefore, a detailed image-level evaluation is provided in Section 6.1 to complement the findings presented here.

### 5.1. Quantitative Detection Evaluation

In this section, we show the results of the YOLO-NAS detector on the original and enhanced images of CUPDD and RUOD. We call YOLO-NAS models that are trained on the original images *original detectors*, and YOLO-NAS models that are trained on enhanced images *domain detectors*. On the one hand, the performance of the 10 YOLO-NAS original and domain detectors on the CUPDD dataset is shown in Table 4. The images of CUPDD are severely degraded with very low visibility, explaining the low overall performance of the original and the domain detectors, where the original detector achieved the highest mAP by a notable margin at 0.38. The leading performance of the original detector was evident in the Bushy class but not in the Leafy class, where it was outperformed by the AutoEnh domain detector by a notable margin of 3%. This is possibly due to the fact that the Bushy class has a more complex shape and edges that are obscured by enhancement noise compared to the simpler Leafy class. Unexpectedly, PCDE achieves very close overall performance to the original detector, although we have discussed in Section 4 how PCDE produces very low-quality and non-visually pleasing images. On the contrary, ICSP has a severely deteriorated mAP performance at 0.22, aligning with the severe deterioration in its enhancement performance. ACDC and TEBCF showed a lower Leafy class mAP at 0.22 and have overall scores of 0.34 and 0.33, respectively. BayesRet showed consistently lower performance across all classes with an overall mAP of 0.30. AutoEnh achieved the highest performance in the Leafy class at 0.31 and an overall score of 0.37. Semi-UIR provided a consistent detection score across all classes with an overall mAP of 0.34, matched by TUDA. Finally, USUIR stood out in the Tapey class at an mAP of 0.40 and achieved an overall mAP of 0.35.

On the other hand, Table 5 shows the detection performance on the RUOD dataset with similar results to Table 4 where the original detector again outperformed all other domain detectors, apart from AutoEnh which had a matching overall performance. Other models achieved moderate performances, including ACDC, TEBCF, BayesRet, and USUIR, which showed reasonably consistent mAP scores across most classes, resulting in overall mAP values between 0.59 and 0.61. Conversely, ICSP consistently ranked with the lowest scores across nearly all classes, yielding the lowest overall mAP at 0.55. There is a noticeable trend where all detectors perform best in the Diver and Cuttlefish classes, often achieving mAP scores above 0.70. Those two classes are mostly found in clear and high-quality images and have a relatively larger instance size than other classes. An observable note is that the Jellyfish class achieved values with the TUDA and BayesRet domain detectors around 3% higher than the original detector. These observations signify the variability in the detection performance using different enhancement methods and across different classes. In addition, no domain detector was able to outperform the original detector, indicating a negative overall effect of enhancement on detection at the dataset level. However, this general conclusion might not hold true on an individual level, where enhancement could have a positive effect on some images and a negative effect on others, resulting in a negative overall performance. Therefore, a more granular analysis that addresses the detection performance before and after enhancement at the image level is required.

These results reflect the aggregate effect of enhancement but do not indicate whether performance improves or declines for specific images, motivating the more detailed per-image analysis.

### 5.2. Qualitative Detection Evaluation

**Figure 6:**
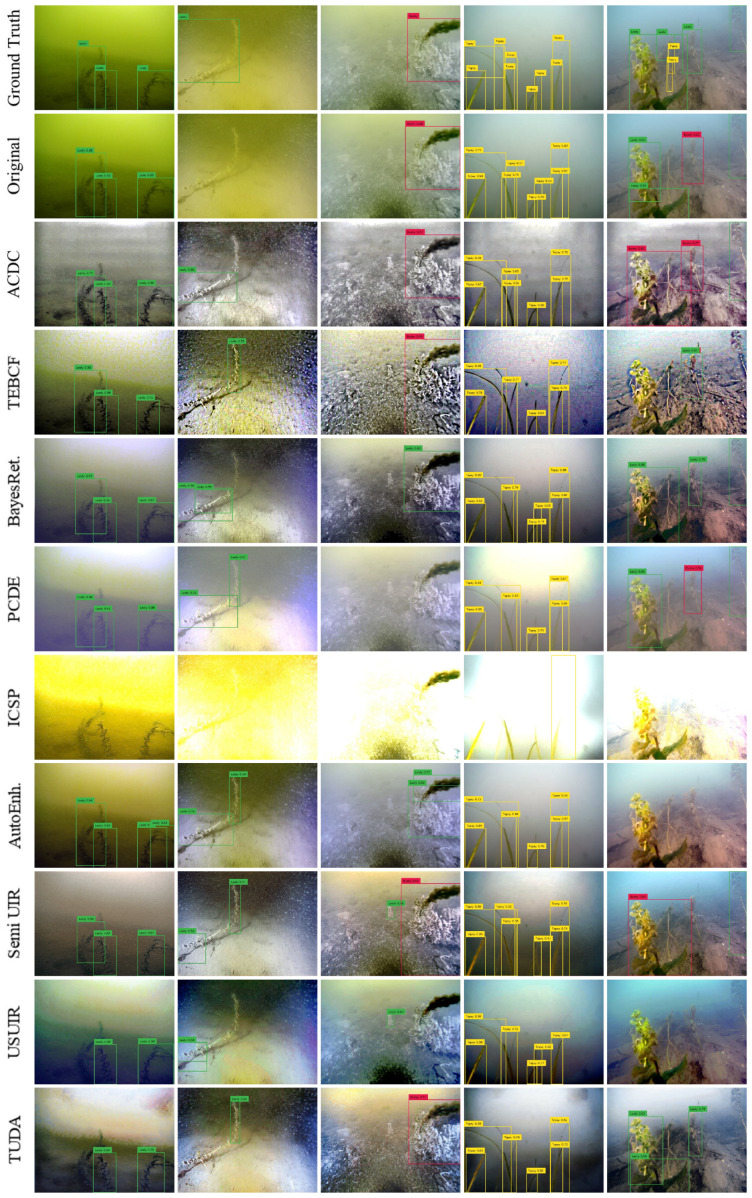
Inference visualization of the original and domain detectors of five random images on the CUPDD dataset.

**Figure 7:**
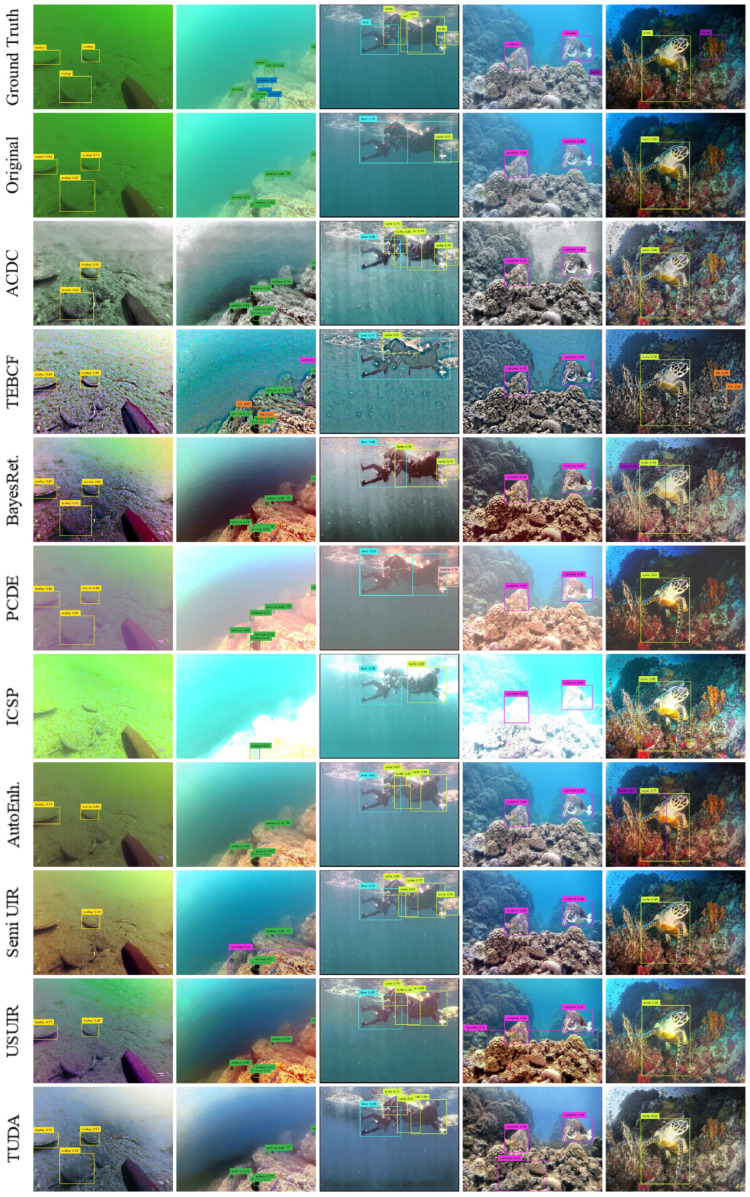
Inference visualization of the original and domain detectors of five random images on the RUOD dataset.

The inference from the original and domain detectors on five random testing images from both CUPDD and RUOD is provided in Figure 6 and Figure 7. For a more convenient evaluation, we opt for a confidence threshold of 0.5. On the one hand, the original detector achieved top performance detecting most objects correctly as shown in Figure 6, apart from the second and last images (counting from the left) where some objects were missed resulting in false negatives (FNs) and other objects were confused resulting in false positives (FPs). On the contrary, most domain detectors increased the FPs and FNs. In addition, the true positives (TPs) detected by the domain detectors generally have lower Intersection over Union (IoU) thresholds compared with the original detector, which lowered the mAP_50–95_ performance in Section 5.1. All domain detectors performed fairly well except for PCDE and ICSP, which have very foggy or overexposed images with few details and deformed objects’ edges. We also observed that in the second image, the original detector was not able to detect the leafy plant, but most domain detectors were able to detect at least parts of the leafy plant. This indicates that the detection performance for this image increased after enhancement, revealing the potential of enhancement to improve the detection performance for some images. Furthermore, Semi-UIR and USUIR detected a leafy plant in the third image that is not in the ground truth. We think that human annotators could not see the leafy plant due to the severe degradation of the original image. Therefore, this detected object will be considered an FP despite the fact that it should be a TP.

On the other hand, similar trends are found in Figure 7 of the RUOD dataset where the original detector outperformed domain detectors in most images. Although some models did not produce visually pleasing images, they were still able to achieve good performance such as ACDC. In contrast, some models that did produce visually pleasing images underperformed compared with other domain detectors. Similar to our observation on CUPDD, we observed some cases where domain detectors outperformed the original detector, such as the case for the third image. We also observe that domain detectors that produce more noise and artifacts have a relatively higher number of FPs compared to other models, such as the case of the TEBCF model. We conclude that the overall negative impact of enhancement could be attributed to diffused edges and increased noise after enhancement. Furthermore, visually pleasing images do not necessarily lead to higher detection performance. In addition, image enhancement might improve the detection performance of some images and might also help in revealing hidden objects in the original images for human annotators.

Note that the confidence threshold of 0.5 used for visualization was chosen to produce clean, readable overlays; quantitative mAP is always computed using the evaluation thresholds described in Section 3 (0.001 for YOLO-NAS, 0.05 for RetinaNet/Faster R-CNN) together with their respective NMS settings.

### 5.3. Enhancement–Detection Metrics Correlation

**Figure 8:**
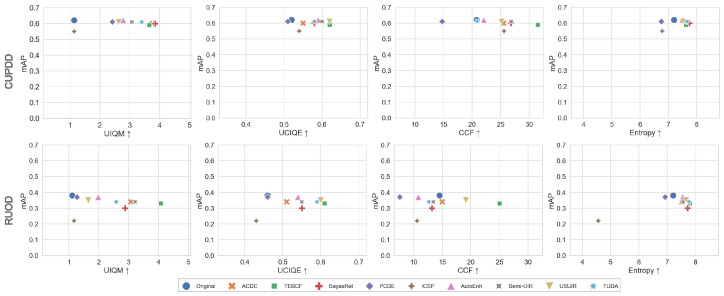
Scatter plots of the selected enhancement metrics against the mAP from the original and domain detectors for the CUPDD and RUOD datasets.

In this section, we generate scatter plots of the mean values of the selected enhancement metrics for each enhanced dataset and the mAP of the domain detectors for both the CUPDD and RUOD as shown in Figure 8. The prevailing noticeable trend in Figure 8 across all metrics is that increases in enhancement metric values do not correlate with increases in mAP. For example, the mAP maintained similar values with the increase in UIQM for CUPDD, whereas it slightly decreased with the increase in UIQM in RUOD. Furthermore, some enhancement models, such as AutoEnh and PCDE, achieved lower CCF scores on RUOD compared to the original images, yet their images performed similarly in terms of the mAP. In contrast, TEBCF and USUIR achieved a higher CCF but lower mAP compared to the original images. The ICSP model is an outlier on most plots and gained lower enhancement and mAP values due to its extremely overexposed images. The scatter plots presented in Figure 8 do not show any consistent relationship between quality and detection metrics with mixed and random performances across different models. This means that the enhancement performance does not reliably predict the detection performance. This discrepancy between the quality and detection metrics might be caused by the sensitivity of current quality metrics, resulting in values that do not accurately reflect the true visual or structural quality of the images. Such outcomes may also be attributed to the fundamental differences between human and machine perception, further emphasizing the need for metrics that simultaneously account for visual quality from a human perspective and detection performance from a machine perspective.

## 6. Per-Image Analysis and Mixed-Set Upper Bound

The analyses in Section 4 and Section 5 reveal a nuanced interaction between image enhancement and underwater object detection. Enhancement affects images heterogeneously: some images benefit substantially, while others degrade, and dataset-level averages fail to capture this variability. To resolve these inconsistencies, this section introduces a principled, image-level evaluation framework that quantifies the impact of enhancement on a per-image basis and establishes an upper bound on the performance achievable through selective enhancement. This analysis forms the core contribution of our work and directly follows the evaluation workflow shown in Figure 1.

### 6.1. Per-Image mAP Protocol

Dataset-level mAP aggregates detections across thousands of images, which obscures the fact that enhancement may benefit some images while degrading others. To capture these image-specific effects, we compute a per-image mAP_50–95_ score for each image and each of its enhanced variants. This mirrors the standard COCO evaluation protocol but is applied at the single-image level.

For a given image, all predicted bounding boxes (for a specific enhancement method or the original version) are first sorted by confidence. A precision–recall (PR) curve is constructed using only the predictions and ground-truth boxes corresponding to that image. True positives and false positives are determined using the same IoU thresholds as in the dataset-level evaluation (0.50 to 0.95 in increments of 0.05). The per-image mAP is the average area under the PR curve across these IoU thresholds. This procedure produces ten well-defined detection scores for each original image (one for the original version and nine for the enhanced versions). These scores allow a fine-grained comparison of enhancement impact at the image level.

**Figure 9:**
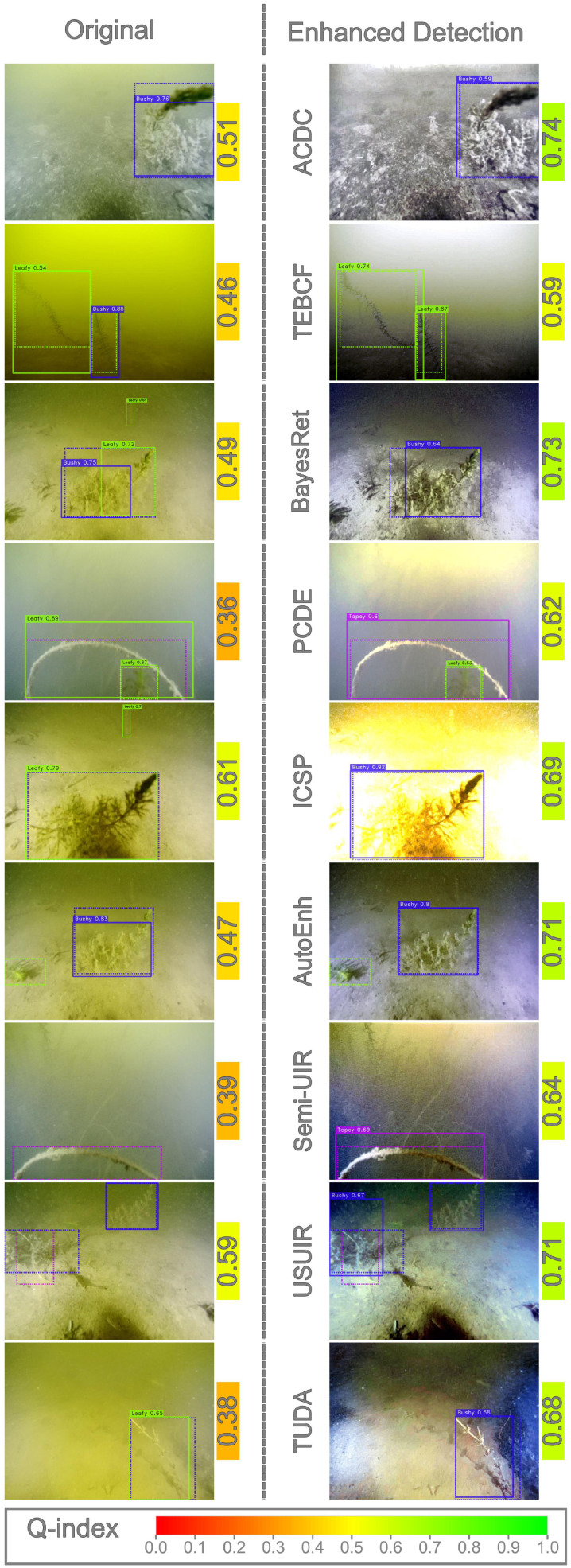
Cases where domain detectors performed better than the original detector on the CUPDD dataset. The ground truth bounding boxes are visualized on each image as dotted bounding boxes. The color-mapped values next to images represent Q-index.

**Figure 10:**
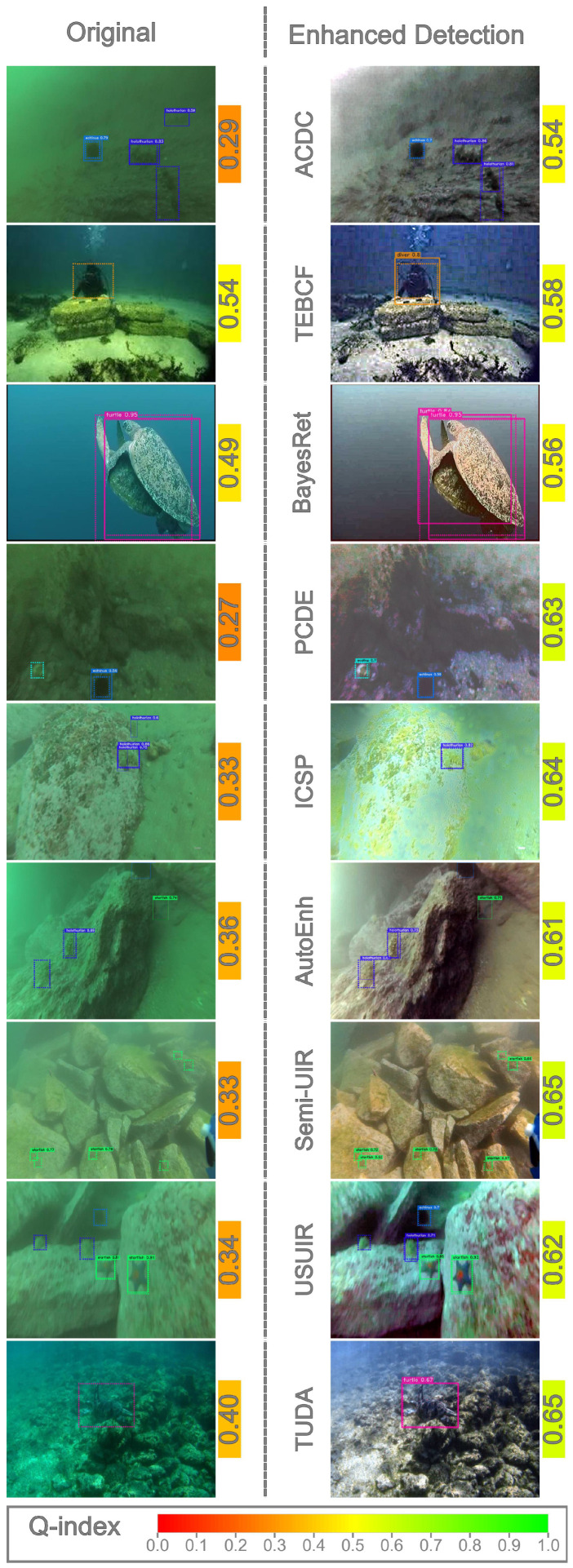
Cases where domain detectors performed better than the Original detector on RUOD dataset. The ground-truth bounding boxes are visualized on each image as dotted bounding boxes. The color-mapped values next to images represent Q-index.

To investigate detection performance at the image level, we visually examined samples from both datasets and compared the predictions produced by the original detectors with those generated by detectors trained on enhanced images (domain detectors). This analysis focuses on cases in which a domain detector outperformed the original detector. Representative examples from all enhancement methods were selected to illustrate the positive impact of enhancement, as shown in Figure 9 and Figure 10. Ground-truth annotations are depicted using dotted boxes, while predicted detections are shown with solid boxes.

Across both datasets, improvements were primarily concentrated in low- to medium-quality images, typically with Q-index values below 0.5. As discussed in Section 4.2, all enhancement algorithms increased the Q-index of these images. High-quality images rarely appeared among the improved cases, suggesting that enhancing already high-quality images may degrade subsequent detection performance.

In the CUPDD dataset (Figure 9), enhancement models exhibited diverse behaviors. ACDC increased contrast and removed color cast, resulting in visually less natural images but producing tighter detection boxes. TEBCF, BayesRet, and TUDA improved object boundaries, converting some false positives into true positives. Surprisingly, ICSP and PCDE, despite occasionally generating overexposed or hazy outputs, also produced instances of improved detection. Methods such as AutoEnh, Semi-UIR, and USUIR frequently revealed objects that the original detector failed to detect.

Similar trends appear in the RUOD dataset (Figure 10). Some methods corrected color distortions while introducing other degradations, yet still improved detection performance. For example, TEBCF removed the strong color cast but added noise while still detecting the diver class, likely due to its distinctiveness. TUDA produced visually clear images that facilitated better foreground–background separation.

Collectively, these observations show that enhancement can improve detection performance under specific conditions, particularly by reducing the disparity between low-quality test samples and higher-quality training samples. These findings extend prior conclusions [11,12,13,16], which mainly reported negative effects of enhancement at the dataset scale. Our results demonstrate that these negative effects do not necessarily apply at the image level. Furthermore, most improved cases fall into low-Q-index bins, indicating that selectively enhancing low-quality images may yield better results than enhancing entire datasets uniformly.

### 6.2. Mixed-Set Construction

While the per-image evaluation reveals when enhancement is beneficial, it also enables a principled method for quantifying the maximum achievable performance that any enhancement–detection pipeline could obtain. Since some images perform best in their original form while others benefit from specific enhancement algorithms, applying enhancement uniformly across an entire dataset is inherently suboptimal. Instead, an ideal system would selectively choose, for each image, whether enhancement should be applied and which enhancement model is most beneficial.

To approximate this ideal behavior, we construct a mixed set that contains, for every image, the version—original or enhanced—that yields the highest per-image mAP. This mixed set represents an upper bound on what a selective enhancement strategy could achieve if it could perfectly predict, in advance, which version of an image leads to the best detection results.

**Figure 11:**
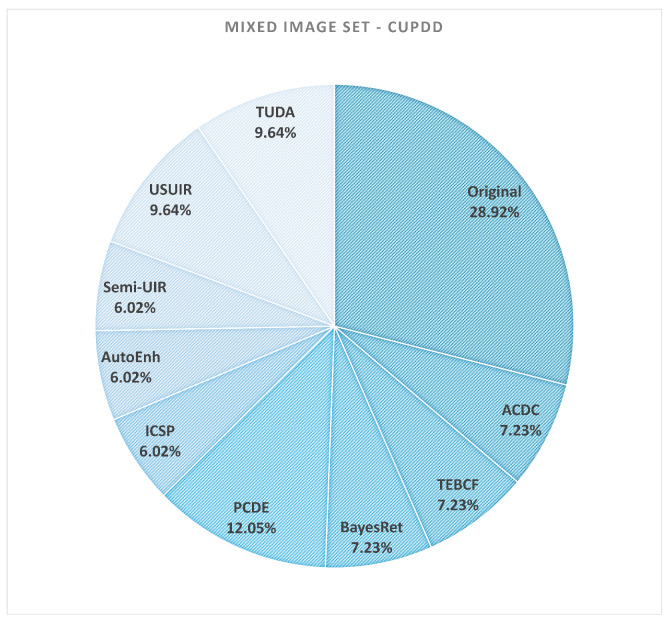
Pie chart of the generated mixed-image set comprising original and enhanced images from the CUPDD test set [54]. The images of this set are selected based on their mAP performance.

**Figure 12:**
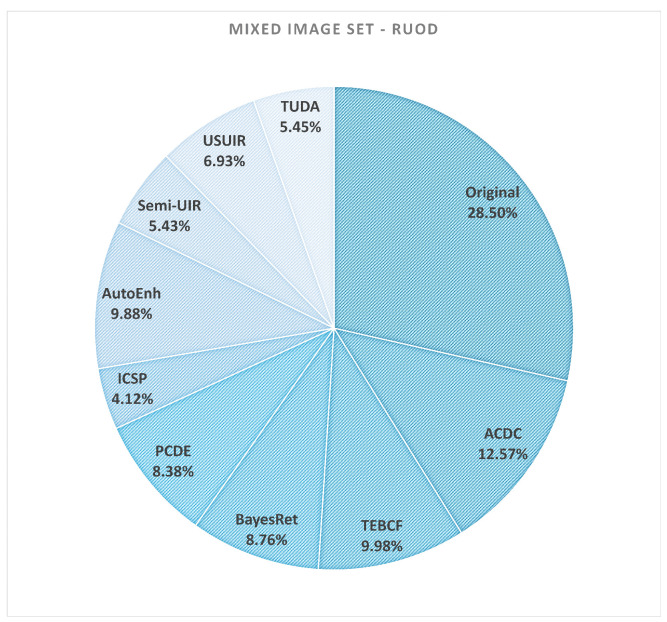
Pie chart of the generated mixed-image set comprising original and enhanced images from the RUOD test set [12]. The images of this set are selected based on their mAP performance.

The composition of the mixed sets for CUPDD and RUOD is visualized in Figure 11 and Figure 12. These pie charts indicate how frequently each enhancement method contributes the highest-performing version of an image. Interestingly, the original images constitute the largest fraction of the mixed sets for both datasets, confirming that enhancement is often unnecessary—and can even be detrimental—for images that are already of high quality.

However, several enhancement methods contribute non-negligible portions (typically 4–12%), demonstrating that enhancement does meaningfully improve detection for specific image conditions, particularly under severe degradation. This reinforces the central insight of our study: enhancement is most effective when applied selectively rather than uniformly.

The mixed set, thus, serves two essential purposes. First, it quantifies the true potential of enhancement when optimally deployed. Second, it provides a benchmark for future research on predictive or learning-based selective enhancement approaches, which aim to approximate the oracle-like behavior embodied in $S_{\mathrm{mix}}$.

### 6.3. Upper-Bound Conclusions

The mixed-set results provide a clear and quantitative answer to a question that has remained ambiguous in prior underwater vision research: *Can underwater image enhancement improve object detection performance?* At the dataset scale, the answer appears to be mostly negative. However, the per-image evaluation and mixed-set construction reveal a fundamentally different reality.

As shown in Table 6, the mixed sets substantially outperform both the original image sets and all individually enhanced sets. On the CUPDD dataset, the average per-image mAP increases from 0.41 (original) to 0.64 (mixed), a relative improvement of 56%. On RUOD, performance increases from 0.68 to 0.77, a relative improvement of 13%. These gains represent recoverable detection performance that is currently lost when enhancement is applied uniformly. In other words, enhancement can improve detection—but only when applied selectively and only for the images that genuinely benefit.

The composition of the mixed sets (Figure 11 and Figure 12) further strengthens this conclusion. The majority of images retain their original form, while several enhancement models contribute meaningfully to the improved subset. This confirms that enhancement is a targeted remedy, not a universal solution. High-quality images rarely benefit from enhancement, and uniform preprocessing tends to degrade their performance. Conversely, severely degraded images—particularly those with low Q-index values—are the primary beneficiaries of enhancement.

Taken together, these findings lead to three key insights:

- Enhancement holds significant but hidden value: Although dataset-level analyses often show performance declines, substantial improvements are achievable when enhancement is used selectively.
- Uniform enhancement is fundamentally limited: No single enhancement model is optimal for all images, and indiscriminate preprocessing suppresses the benefits that enhancement can provide.
- Selective enhancement is the correct path forward: The mixed set provides an attainable upper bound that defines the performance target for future enhancement strategies, including adaptive or learning-based approaches capable of predicting which images should—or should not—be enhanced.

Overall, the upper-bound analysis demonstrates that the long-standing perceived conflict between underwater image enhancement and object detection arises not from an inherent incompatibility, but from a mismatch between uniform enhancement strategies and the highly image-dependent nature of underwater visual degradation. By introducing per-image evaluation and mixed-set analysis, this work resolves that contradiction and shows that enhancement can be beneficial when applied selectively. Importantly, the observed upper bound also suggests simple, practical heuristics for real-world deployment, such as triggering enhancement only for low-quality images or for cases where detector confidence is low or detections are sparse. These strategies do not require oracle knowledge, yet are directly motivated by the empirical behavior revealed in this study, providing a realistic pathway toward adaptive enhancement–detection pipelines.

In several cases, the per-image analysis revealed objects that were correctly detected by the models but missed during manual annotation. This highlights a perception gap that can arise in heavily degraded underwater imagery, where visual ambiguity challenges even expert annotators. Such gaps may affect the reliability of existing ground-truth labels and suggest that future underwater benchmarks could benefit from iterative or model-assisted annotation strategies.

## 7. Conclusions

This work presented a rigorous framework for understanding the interaction between underwater image enhancement and object detection, addressing a long-standing gap in the literature. Through quality distribution analysis, detailed per-image mAP evaluation, and mixed-set upper-bound construction, we demonstrated that the effect of enhancement is highly image-dependent rather than uniformly beneficial or harmful. Enhancement consistently improves detection on low-quality images suffering from severe underwater degradation while often degrading performance on high-quality images. These findings explain why prior studies—based solely on dataset-level averages—have reported conflicting conclusions, and they underscore the necessity of evaluating enhancement at the appropriate granularity.

By selecting, for each image, the version that achieves the highest per-image mAP, the mixed-set formulation revealed substantial recoverable performance gains: a 56% improvement on CUPDD and a 13% improvement on RUOD. These gains represent the true potential of enhancement when applied selectively rather than uniformly. The results provide a clear path forward: adaptive, content-aware, or learning-based selection strategies that decide when and how to apply enhancement are likely to outperform both raw detectors and any single enhancement model. Rather than viewing enhancement and detection as competing processes, our study shows that they can be highly complementary when integrated intelligently, offering new insights and opportunities for the next generation of underwater vision systems.

## Figures and Tables

**Table 1:** Quantitative evaluation of the selected enhancement models using the mean and standard deviation (std) values of the UIQM [55], UCIQE [56], CCF [57], and Entropy [58] on the CUPDD [54] dataset. Highest scores are highlighted in bold.

CUPDD [54] Dataset								
Models	UIQM ↑		UCIQE ↑		CCF ↑		Entropy ↑	
	Mean	Std.	Mean	Std.	Mean	Std.	Mean	Std.
Original	1.11	0.35	0.46	0.03	14.52	4.23	7.21	0.28
ACDC [5]	3.07	0.54	0.51	0.02	14.97	2.93	7.52	0.11
TEBCF [26]	**4.07**	0.69	**0.61**	0.02	**25.03**	5.01	**7.80**	0.09
BayesRet [25]	2.88	0.66	0.55	0.03	13.17	3.86	7.72	0.05
PCDE [6]	1.27	0.31	0.46	0.02	7.55	1.42	6.93	0.28
ICSP [29]	1.17	0.40	0.43	0.04	10.57	3.04	4.56	0.96
AutoEnh. [7]	1.98	0.57	0.54	0.03	10.79	2.53	7.55	0.19
Semi UIR [32]	3.21	0.59	0.55	0.04	13.42	4.12	7.56	0.19
USUIR [33]	1.65	0.42	0.60	0.02	19.13	4.35	7.67	0.15
TUDA [34]	2.58	0.56	0.59	0.02	12.62	2.33	7.77	0.07

**Table 2:** Quantitative evaluation of the selected enhancement models on the RUOD [12] dataset using the mean and standard deviation (std) values of the UIQM [55], UCIQE [56], CCF [57], and Entropy [58]. Highest scores are highlighted in bold.

Models	UIQM ↑		UCIQE ↑		CCF ↑		Entropy ↑	
	Mean	Std.	Mean	Std.	Mean	Std.	Mean	Std.
Original	1.14	1.18	0.52	0.05	20.79	6.56	7.20	0.36
ACDC [5]	3.76	0.76	0.55	0.03	25.49	5.02	7.67	0.16
TEBCF [26]	3.65	0.94	**0.62**	0.03	**31.50**	4.29	7.62	0.23
BayesRet [25]	**3.85**	0.80	0.58	0.03	26.80	7.15	**7.74**	0.12
PCDE [6]	2.42	0.75	0.51	0.04	14.76	3.72	6.75	0.43
ICSP [29]	1.14	1.41	0.54	0.05	25.59	7.87	6.78	0.77
AutoEnh [7]	2.78	1.17	0.59	0.04	22.05	5.93	7.48	0.31
Semi UIR [32]	3.07	1.17	0.60	0.04	26.84	8.36	7.60	0.22
USUIR [33]	2.63	0.84	**0.62**	0.04	25.16	7.17	7.53	0.26
TUDA [34]	3.40	0.95	0.58	0.02	20.88	4.52	7.65	0.17

**Table 3:** Comparison of the detection performance of different object detection algorithms on the original images of the RUOD dataset [12] using the mean Average Precision (mAP). Highest scores are highlighted in bold.

Methods	mAP_50_	mAP_50–95_
YOLO-NAS [52]	**0.85**	**0.62**
RetinaNet [53]	0.82	0.56
Faster R-CNN [53]	0.81	0.52

**Table 4:** Per-class detection evaluation of the original and enhanced CUPDD [54] dataset by the selected enhancement models using the mean Average Precision (mAP_50–95_). Highest scores are highlighted in bold.

Methods	Bushy	Leafy	Tapey	mAP
Original	**0.46**	0.29	**0.40**	**0.38**
ACDC [5]	0.42	0.22	0.37	0.34
TEBCF [26]	0.40	0.22	0.36	0.33
BayesRet [25]	0.30	0.23	0.36	0.30
PCDE [6]	0.43	0.30	0.39	0.37
ICSP [29]	0.28	0.16	0.23	0.22
AutoEnh [7]	0.43	**0.31**	0.37	0.37
Semi UIR [32]	0.38	0.23	**0.40**	0.34
USUIR [33]	0.40	0.25	**0.40**	0.35
TUDA [34]	0.38	0.27	0.38	0.34

**Table 5:** Per-class detection evaluation of the original and enhanced RUOD [12] dataset by the selected enhancement models using the mean Average Precision (mAP_50–95_). Highest scores are highlighted in bold.

Methods	Holothurian	Echinus	Scallop	Starfish	Fish	Corals	Diver	Cuttlefish	Turtle	Jellyfish	mAP
Original	**0.50**	**0.50**	**0.51**	**0.55**	**0.55**	**0.54**	**0.75**	**0.85**	**0.85**	0.59	**0.62**
ACDC [5]	0.48	0.48	0.48	0.53	0.52	0.51	0.73	0.83	0.84	0.60	0.60
TEBCF [26]	0.48	0.49	0.49	0.53	0.52	0.49	0.71	0.81	0.81	0.58	0.59
BayesRet [25]	0.48	0.47	0.49	0.53	0.53	0.52	0.72	0.83	0.84	**0.62**	0.60
PCDE [6]	0.48	0.49	**0.51**	0.53	0.54	**0.54**	0.73	0.84	**0.85**	0.59	0.61
ICSP [29]	0.43	0.49	0.42	0.48	0.48	0.47	0.70	0.76	0.79	0.52	0.55
AutoEnh [7]	0.49	**0.50**	**0.51**	0.54	0.54	**0.54**	0.74	**0.85**	**0.85**	0.59	**0.62**
Semi UIR [32]	0.47	0.49	0.48	0.53	**0.55**	0.52	0.73	0.84	0.84	0.61	0.61
USUIR [33]	0.47	0.49	0.49	0.53	0.54	0.53	0.73	0.84	**0.85**	0.60	0.61
TUDA [34]	0.47	0.48	0.47	0.53	**0.55**	0.52	0.73	0.84	0.84	**0.62**	0.61

**Table 6:** Per-image mean Average Precision (mAP_50–95_) evaluation of the original, enhanced, and mixed image sets. The values shown represent the average of the individual mAP for each image in a set. Highest scores are highlighted in bold.

	Set	Per-Image mAP	
		CUPDD	RUOD
	Original	0.41	0.68
Enhanced	ACDC [5]	0.36	0.66
	TEBCF [26]	0.35	0.66
	BayesRet [25]	0.35	0.66
	PCDE [6]	0.39	0.67
	ICSP [29]	0.26	0.61
	AutoEnh [7]	0.37	0.68
	Semi UIR [32]	0.37	0.67
	USUIR [33]	0.37	0.67
	TUDA [34]	0.38	0.67
	Mixed (CUPDD)	**0.64**	**-**
	Mixed (RUOD)	**-**	**0.77**

## Data Availability

The data presented in this study are openly available in Enhancement-Detection-Analysis at https://github.com/RSSL-MTU/Enhancement-Detection-Analysis (accessed on 24 December 2025).
